# Pulmonary Tuberculosis Diagnosis Using an Intelligent Microscopy Scanner and Image Recognition Model for Improved Acid-Fast Bacilli Detection in Smears

**DOI:** 10.3390/microorganisms12081734

**Published:** 2024-08-22

**Authors:** Wei-Chuan Chen, Chi-Chuan Chang, Yusen Eason Lin

**Affiliations:** 1 Division of Teaching and Education, Teaching and Research Department, Kaohsiung Veterans General Hospital, Kaohsiung 813414, Taiwan; wcchen1027@vghks.gov.tw (W.-C.C.);; 2 Department of Pharmacy Master Program, Tajen University, Yanpu 907101, Taiwan; 3 Graduate Institute of Human Resource and Knowledge Management, National Kaohsiung Normal University, Kaohsiung 802561, Taiwan

**Keywords:** TB smear, AI, machine learning, TB diagnosis

## Abstract

Microscopic examination of acid-fast mycobacterial bacilli (AFB) in sputum smears remains the most economical and readily available method for laboratory diagnosis of pulmonary tuberculosis (TB). However, this conventional approach is low in sensitivity and labor-intensive. An automated microscopy system incorporating artificial intelligence and machine learning for AFB identification was evaluated. The study was conducted at an infectious disease hospital in Jiangsu Province, China, utilizing an intelligent microscope system. A total of 1000 sputum smears were included in the study, with the system capturing digital microscopic images and employing an image recognition model to automatically identify and classify AFBs. Referee technicians served as the gold standard for discrepant results. The automated system demonstrated an overall accuracy of 96.70% (967/1000), sensitivity of 91.94% (194/211), specificity of 97.97% (773/789), and negative predictive value (NPV) of 97.85% (773/790) at a prevalence of 21.1% (211/1000). Incorporating AI and machine learning into an automated microscopy system demonstrated the potential to enhance the sensitivity and efficiency of AFB detection in sputum smears compared to conventional manual microscopy. This approach holds promise for widespread application in TB diagnostics and potentially other fields requiring labor-intensive microscopic examination.

## 1. Introduction

Tuberculosis is treatable, preventable, and curable. Sustained declines in tuberculosis deaths in many countries during the past 50 years provide evidence that ending the pandemic is foreseeable [1]. However, tuberculosis, which has plagued humanity and has killed hundreds of millions of people over the past two centuries, remains a global public health threat. In 2023, 1.3 million people died from tuberculosis (95% UI: 1.18–1.43 m), including 167,000 people with HIV, representing more deaths than any other infectious disease [2]. World leaders in the most recent United Nations High-Level Meeting (UNHLM) on TB made commitments and requests to address the global tuberculosis crisis [1], which included providing comprehensive care to all people with TB, addressing the crisis of drug-resistant TB, strengthening the engagement of civil society and communities affected by TB, and enabling and strengthening TB research. It highlights the need for comprehensive care, addressing drug-resistant TB, engaging civil society and communities, and promoting TB research. The commitments made during the meeting provide a strong impetus to accelerate the TB response and work towards ending TB.

The World Health Organization (WHO) recommends the acid-fast stain method of sputum smears as the most robust and economical method for the first line of laboratory diagnosis of pulmonary tuberculosis. This method relies on the microscopic examination of sputum samples for acid-fast mycobacteria bacilli (AFB). However, it is important to note that the sensitivity and specificity of smear microscopy are poor, as it only detects 10 to 75% of pulmonary TB cases. Additionally, smear microscopy is labor-intensive and tedious. While new molecular-based methods like Xpert MTB/RIF have become available, they have not been widely deployed in rural areas due to substantially higher costs and infrastructure constraints, which may not be affordable in many countries with high TB burden in the foreseeable future [3,4]. Thus, despite advancements in molecular diagnostics, the acid-fast stain method remains the recommended first-line laboratory diagnosis for pulmonary tuberculosis due to its robustness and cost-effectiveness [5,6,7]. The WHO suggests the availability of 1.1 microscopy laboratories per every 100 thousand population to enhance the diagnostic capacity for tuberculosis [2].

The situation of unsatisfactory TB smear accuracy (sensitivity and specificity) seems to be the same scenario as pathology. For more than 100 years, pathologists have relied on manual microscopy, the same as microbiologists, for laboratory diagnosis. It seemed unsolvable until the development of artificial intelligence (AI), image recognition, and machine learning algorithms in the late 1990s. Machine learning (ML) is based on artificial neural networks, which mimic human brain processes by passing data through hidden layers by connected neurons, with the output layer providing the estimation or prediction. The key advantage of ML is its ability to automatically extract features of the input information from the iteration of calculation back and forth among input layers, the hidden layers, and the output layer(s). These developments have motivated several large clinical trials to use ML technologies in pathology. Digital pathology has become a trending movement in the so-called “Smart Hospitals”, where pathology specimens can be digitalized, electronically transferred, diagnosed, reviewed, and the report issued. However, no “Digital Microbiology” products and services have been developed accordingly.

Recently, some automated TB smear microscopy systems have been developed that take advantage of artificial intelligence (AI) and big data analysis, which may significantly increase the sensitivity of TB smear microscopy [8,9,10,11,12,13,14,15,16,17,18,19,20,21]. However, most of the research focused on algorithms and deep learning model building, with less focus on system integration (e.g., evaluation of hardware and software together). Such an integrated system may include a motorized stage to load the smear slides into a bright-field microscope (hardware). Then, the system performs auto-focus, digitally captures the smear images, analyzes the images, and classifies smear slides as positive or negative (software). Although all these studies reported better performance than human examination, most are still in development or just “proof-of-concept” systems. Until 2022, an integrated microscopic system was commercialized for automatic detection of AFB, which has received medical device registration in several countries [22,23]. This is a continuation study to describe the performance characteristics and medical technician’s workload of a diagnostic algorithm for the identification of AFB under a microscope using image recognition technology.

## 2. Materials and Methods

Study Hospital: The Study Hospital was formerly an infectious diseases specialty hospital located in Southern Jiangsu, China. The hospital has 900 beds, of which, 210 are in the respiratory department. An average of 80 smears are tested for mycobacteria in the laboratory. At least three technicians are on duty daily to perform TB smear microscopy. All specimens in the study were processed by liquid-base culture method for MTB identification.

Specimen: This study initially included 1150 smears. One hundred fifty smears were rejected due to incomplete stain removal (n = 60), smear location shift (n = 8), smear being too thick (n = 3), smear being too thin (14), smear dropped off (n = 4), and slide size too big or too small for the system (n = 21). The remaining 1000 smears were enrolled.

Smear Stain: We followed the standardized protocol for the modified Kinyoun acid-fast stain of smears. All specimens were processed with N-acetyl-L-cysteine-sodium hydroxide for decontamination. A smear was then made by spreading a thin layer of the processed sputum sample onto a glass slide and allowing it to air dry, followed by heat fixation 2 to 3 times. Then, the slide was flooded with Kinyoun carbolfuchsin stain for 5 min after 1 min with absolute methanol for 1 min. The slide was rinsed briefly (3 to 5 s) with 50% ethanol, followed by water rinsing. Moreover, the slide was decolorized with 1% sulfuric acid for 2 min or until no more color runs from the slide. Afterward, the slide was rinsed with water, counterstained with methylene blue for 1 min, and rinsed to air dry. After the technicians examined the smears with a manual microscope and issued the test results in the laboratory report, the same set of slides was transferred to another technician for comparison by the automated system.

Image Recognition Model for TB Bacillus: The machine learning model applied in this study for TB bacilli detection utilizes a hybrid approach, combining supervised and unsupervised learning algorithms. The model building started with supervised learning, where the system is trained to identify candidate TB objectives based on their morphological characteristics. The convolutional neural network (CNN), a class of deep learning models particularly effective in image recognition tasks, was trained on more than 100,000 TB smears, learning to distinguish the distinctive bacillus-shaped morphology of acid-fast bacilli (AFB) from other cellular debris and artifacts in the sputum smear, similar to approaches used in other medical imaging applications [24]. Once potential bacilli were identified, the model transitions to an unsupervised learning phase. This stage employed another CNN model to refine the classification. The unsupervised model is exposed to a diverse set of image objects containing TB-positive and TB-negative bacilli with labels by medical technicians. Through this process, the model learns to identify subtle features and patterns that distinguish positive TB bacilli from other similar-looking objects. This unsupervised approach is particularly valuable as it allows the model to discover complex, non-linear relationships in the data that might not be apparent to human observers or easily codified in rule-based systems. Following the unsupervised learning phase, the model undergoes a refinement step where the image objects classified as TB-negative by the unsupervised model are removed from further consideration. This step effectively prunes the candidate pool, leaving only the objects that the model considers highly likely to be TB bacilli. This refinement process significantly enhances the model’s precision, reducing the likelihood of false positives in the final output, a technique that has shown promise in other medical imaging applications [25]. The overall architecture of this hybrid model allows for continuous improvement and adaptation. As new data become available, both the supervised and unsupervised components can be fine-tuned, enhancing the model’s performance over time. The use of deep learning techniques, particularly in the unsupervised phase, enables the model to capture complex, high-dimensional features that may not be apparent to human observers, potentially leading to improved sensitivity compared to traditional manual microscopy.

Procedures for a Parallel Study: An automated intelligent medical microscope system (“system”) (TB-Scan, Wellgen Medical, Kaohsiung, Taiwan) was installed in a negative pressured isolation laboratory. The system consists of two components: (1) microscopic imaging acquisition hardware with auto-focusing and slide-scanning capability to cover the 1 cm by 2 cm specimen area based on WHO recommendation (300 fields @1000× oil lens); (2) an image recognition algorithm for detection and classification of positive AFBs. The image acquisition hardware is designed to refocus every field of view to overcome the inconsistency of smear thickness. This procedure ensures that each image acquired is on focus, though the total acquisition time could be longer. After the microscopic images were digitally acquired and stored, candidate AFBs were detected and marked, and the marked bacilli were differentiated from other substances and tissues in the smear based on color and morphological features, as mentioned previously. Such a CNN model was pre-trained with a diverse set of specimen samples, more than 100,000 TB smears from across Asia, mostly coming from Taiwan and partially from China, India, and Japan, to minimize the potential overfitting problem [22,23]. The results were recorded as positive if any AFB was identified in the image of the slide. The laboratory technician supervisor served as the Gold Standard in evaluating the system’s performance. The whole system was powered and controlled by a custom-made internal personal computer (CPU: Intel Gen10 Core i7-10700TE w/16 GB RAM and an Nvidia GTX 1650 GPU w/4 GB DDR5, Santa Clara, CA, USA).

Quality Control: All positive smears detected by TB-Scan were re-examined by a microscope (Olympus CX-21) under a 1000× oil lens for verification, and microscopic images were captured and stored by a cellphone (iPhone 13, Apple Inc., Cupertino, CA, USA).

Data Interpretation: Test performance evaluation is based on sensitivity and specificity. Sensitivity (also called the true positive rate) measures the proportion of positives correctly identified as such (e.g., the percentage of positive TB smears correctly identified from the true positives). Specificity (also called the true negative rate) measures the proportion of actual negatives correctly identified as such (e.g., the percentage of negative TB smears correctly identified as not having the condition). Negative predictive value (NPV) measures the ratio of true negative to all those identified as negative. NPV is an effective indicator for a screening test because its characteristics can predict how likely it is truly negative (e.g., healthy) in case of a negative test result.

## 3. Results

**Figure 1:**
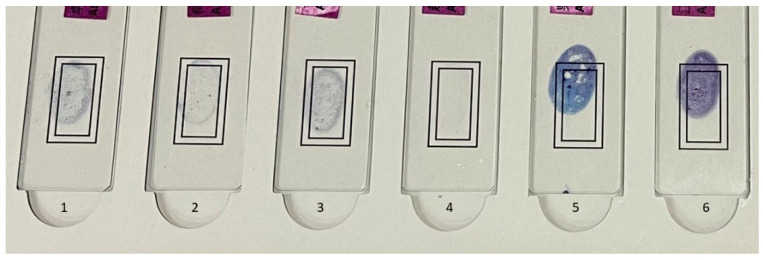
Examples of smear characteristics: #1 through #3 were acceptable. The #4 smear was too thin, and #5 had incomplete stain removal. The #6 smear was too thick, and both #5 and #6 smears were outside of the valid scanning target area.

Specimen Characteristics: Of the 1150 smears for this study, 150 were rejected due to incomplete stain removal (n = 60), smear location shift (n = 8, see Figure 1), smear being too thick (n = 3), smear being too thin (14), smear dropped off (n = 4), and slide size too big or too small for the system (n = 21).

Initial Results from the Automated System: The original hospital clinical records on acid-fast stains indicated that there were 194 AFB-positive smears and 806 AFB-negative smears. Based on TB-Scan’s results, there were 210 AFB-positive smears and 790 AFB-negative smears. Of the 210 AFB-positive smears by TB-Scan, 198 smears contained AFB under microscope examination, and AFB was not found in the remaining 12 smears. Based on the results mentioned above, the confusion matrix is as follows in Table 1.

**Figure 2:**
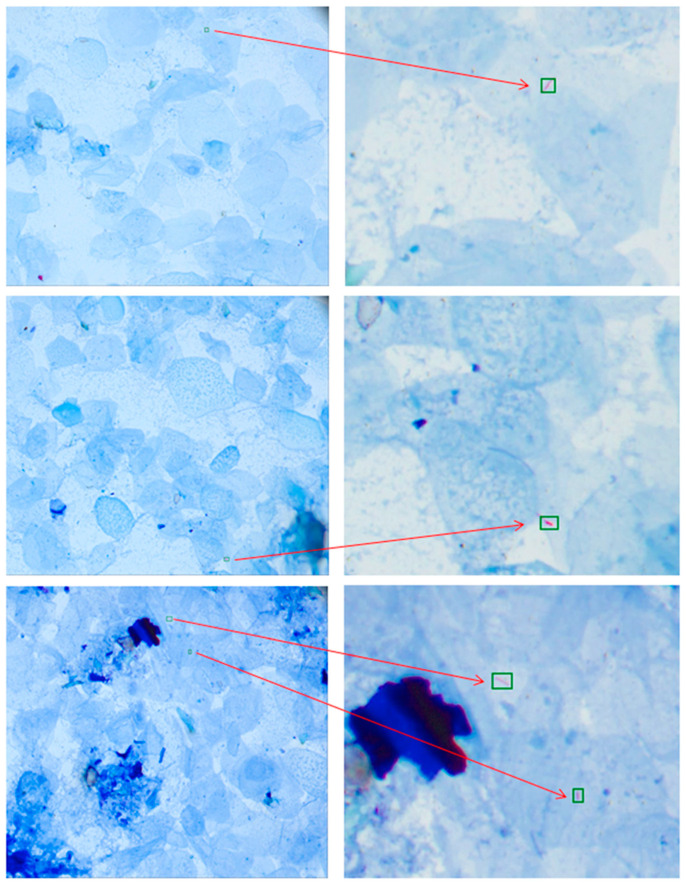
Examples of scanty AFBs detected by the automation system (each image is in 2448 × 2048 resolution with a pixel size of 0.173 μm).

The accuracy was 95.00% (950/1000), with a sensitivity of 91.24% (177/194), specificity of 95.91% (773/806), false negative rate of 8.76% (17/194), and false positive rate of 4.09% (33/806). However, 21 smears that were previously reported as negative were found positive by TB-Scan. The microscopic images demonstrated scanty AFBs (Figure 2).

Discrepancy Resolution and Updated Performance: After presenting the images to the medical technician in the study hospital (our gold standard), the technician ruled out four smears and maintained her judgment as negative, agreeing that the remaining 17 smears should have been recorded as positive. Therefore, the confusion matrix was re-calculated as follows in Table 2.

The accuracy was recalculated as 96.70% (967/1000), with a sensitivity of 91.94% (194/211), specificity of 97.97% (773/789), false negative rate of 8.06% (17/211), and false positive rate of 2.03% (16/789). The negative predictive value (NPV) was 97.8% at a prevalence of 21.1% (211/1000).

Among the 17 smears originally found negative, there were 8 recorded as scanty, 7 as 1+, 1 smear as 2+, and 1 smear as 3+.

User Feedback and Efficiency: During the study, we also interviewed the three laboratory technicians about the system’s user-friendliness and the key benefit to them if they decide to apply such a system in their routine procedures. The technicians’ reports ranged from “above average” (4 points) to “excellent” (5 points), yielding an average of 4.67 points in user-friendliness. The first key comment from the technicians was that the system can help them to eliminate more than 85% of negative smears and only focus on reviewing the remaining 15% of slides. Their average time spent in the manual microscopic examination per smear per person was reduced from 5 min to around 2 min if using the automated system based on a 100-smear workload every day. This is equivalent to a time saving of 5 person-hr per day that the technicians can work on other important laboratory errands while the automated system reads the smears in parallel. Secondly, the technicians were surprised how the image recognition software could detect scanty acid-fast bacilli (Figure 2), which was too difficult for human eyes. Lastly, reading TB smears under microscopy for 4 to 5 h per day is unhealthy for human eyes. The automated system can significantly reduce the eye fatigue associated with manual microscope examination.

One of the key obstacles to applying digital solutions in microscopic systems is the image/data size, which could be a major cost concern. In this study, images generated for each smear covering 300 fields @1000× oil lens is about 60 MB on average, which is significantly smaller than the images generated from the whole slide scanner (WSI), which could be as big as 4 to 6 GB.

## 4. Discussion

The most economical, rapid, and readily available method for laboratory diagnosis of TB is acid-fast staining of sputum smear to identify mycobacterial acid-fast bacilli (AFB). However, the sensitivity of smear microscopy is highly variable [26] due to less experienced or trained staff, long hours of workload, and no presence of quality assurance [27,28,29,30,31]. New technologies, such as the Xpert and TB-LAMP, based on molecular methods, are becoming available. In addition, the fluorescence in situ hybridization (FISH) tests were also used for directly detecting mycobacteria in sputum, which has been successfully implemented in India [32,33,34]. However, it is unlikely that these technologies will be affordable replacements for smear microscopy in many high-burden countries without subsidy from the WHO or Gates Foundations. Thus, if automation, AI, and machine learning can be applied to TB smears, such a system may significantly increase the sensitivity of TB smear microscopy. Then, one may re-evaluate the pros and cons of TB smear microscopy and TB molecular methods, given the trial data that the test sensitivity and specificity are equivalent between TB smear microscopy and TB molecular methods.

In this on-site test, the test system achieved an accuracy of 96.70% (967/1000), sensitivity of 91.94% (194/211), specificity of 97.97% (773/789), false negative rate of 8.06% (17/211), and false positive rate of 2.03% (16/789). The system performed more than 90% in both test sensitivity and specificity, well above previous studies. Due to more consistent specimen preparation, the overall detection performance was better than the previous two studies [22,23]. This is competitive with Xpert, which has a sensitivity of around 90% as well [2]. In addition, regardless of the costs and resource issues with molecular methods, TB smear microscopy continues to play a role in TB diagnosis in monitoring the treatment of TB cases [2,6,7]. It is noteworthy that 17 smears were false negatives based on TB-Scan analysis. After carefully examining each scanned image, both technicians in this study could not find images with AFB. A smear that contains AFB may be outside of the scan area. Thus, to minimize such false negative results, smear preparation should follow a standardized procedure, and the specimen area should be in accordance with TB-Scan’s scan area.

One of the important pieces of feedback from the medical technicians in the study hospitals is that they spent significantly less time, an average reduction of 5 person-hour per work day, in reading the microscope. The literature has documented the health hazards of prolonged microscopic work [35,36,37]. In a study with 450 enrolled study pathologists, 84.8% complained of musculoskeletal disorders (MSD), with the neck being the most common location of pain [35]. Furthermore, 74.8% reported visual refractive errors, among which myopia took the highest place [36]. Another study, with 163 pathologists participating in the study, showed that 40% of responders reported musculoskeletal problems in the previous month [37]. Almost 90% of pathologists had visual refraction errors, mainly myopia [37]. Using the automated system in our study may save three-fifths of the time spent reading smears on the microscope, improving the medical technicians’ work morale. Furthermore, the medical technicians in the study hospital reported that the system usability ranged from “above average” to “excellent”. We would like to credit the company that hired our in-house medical technicians who participated in the user interface design process. It is important that our user interface is compatible with the laboratory’s existing workflow and standard operating procedures as closely as possible, so no ambiguity and confusion occur.

Current guidelines and recommendations state that smear microscopy alone cannot differentiate *Mycobacterium tuberculosis* complex and non-tuberculous mycobacteria (NTM) [2,30,38]. While culture is considered the gold standard diagnostic method for TB due to its high specificity and sensitivity, it is not commonly used due to cost, infrastructure requirements, and the long turnaround time for results [39,40,41]. Huang et al. hypothesized that for performance evaluation of smear microscopy automation systems, the gold standard should be the consensus of expert technicians rather than culture [22]. The rationale behind this hypothesis is that smear microscopy inherently cannot distinguish between *M. tuberculosis* and NTM. Using culture as the gold standard for evaluating smear microscopy automation systems may lead to false negatives when NTM is present, which would be detected as positive by the automation system. Therefore, we support the hypothesis that the performance evaluation of smear microscopy automation systems should use a panel of experienced medical technicians as the reference standard rather than culture. This approach would provide a more accurate and fair assessment of the system’s ability to detect TB bacilli in smear microscopy images, as it aligns with the inherent limitations of smear microscopy in differentiating between *M. tuberculosis* and NTM.

Lastly, when considering the field deployment of an automated microscope system for clinical laboratories, several issues are noteworthy and could be considered as weaknesses: (a) Slide size compatibility: While the slide tray design of the automated system can accommodate most commercial slides, some slides may be too large to fit into the tray slots or too small and prone to falling out of the slide tray. This could impact the system’s ability to process certain slide formats effectively. (b) Stain quality: The quality of the manual staining technique can influence the performance of the automated system, as the recognition software relies on color as an important parameter for detecting acid-fast bacilli (AFB). Inconsistent or suboptimal staining may compromise the system’s ability to accurately identify AFB. We suggest that the use of commercially available automatic stain systems may well resolve the problems. (c) Image size: Most studies use whole slide scanners (WSIs) from digital pathology trying to capture mycobacteria at 400×. Regardless of whether the image quality is acceptable for detecting mycobacteria, the image size could be an issue. The large image size of a WSI, ranging from 4 to 6 GB for the whole 1 cm by 2 cm area, could be costly in data storage and network transfer. In this study, the test system takes each field of view into a compressed image file (i.e., JPEG), and the total data size for one smear is only 60 MB on average, which is a cost advantage for users when considering the digital solution.

## 5. Conclusions

This study demonstrated the potential of an automated intelligent microscopy system incorporating deep learning to improve the diagnosis of pulmonary tuberculosis through the detection of acid-fast bacilli (AFB) in sputum smears. The system achieved an overall accuracy of 96.70%, with a sensitivity of 91.94% and specificity of 97.97%. These results indicate a significant improvement over conventional manual microscopy, particularly in detecting scanty AFB that may be missed by human observers. In addition, the negative predictive value (NPV) of 97.85% at a prevalence of 21.1% is particularly noteworthy. This suggests that the system could be an effective tool for screening, as it reliably identifies negative samples. In resource-limited settings with high TB burdens, this could reduce the workload on laboratory technicians by allowing them to focus their attention on the smaller number of potentially positive samples. The system’s ability to detect previously missed positive cases (17 out of 211 total positives) highlights its potential to improve case detection rates. This is crucial in the global fight against TB, where early and accurate laboratory diagnosis is key to effective treatment and prevention of transmission.

From an operational perspective, the feedback from laboratory technicians regarding the system’s user-friendliness and efficiency improvement is encouraging. The reported time savings of approximately 5 person-hours per day for a 100-smear workload represent a significant improvement in laboratory efficiency. This could allow for increased testing capacity or reallocation of human resources to other critical tasks. Moreover, the reduction in eye strain and fatigue for technicians is an important occupational health benefit that should not be overlooked. Prolonged microscopy work can lead to various health issues, and any system that can alleviate this burden is valuable. Furthermore, the relatively small file size of the digital images (average 60 MB per smear) compared to whole slide imaging (WSI) systems is another advantage. This makes the system more feasible for implementation in resource-limited settings where data storage and transfer capabilities may be constrained.

However, it is important to note this study’s limitations. The rejection of 150 smears due to various quality issues highlights the need for standardized sample preparation and potential training requirements for optimal system use. Future studies should address these issues and explore ways to minimize sample rejection rates.

While the performance of this system is promising, it is crucial to consider its place within the broader context of TB diagnostics. Molecular methods like Xpert MTB/RIF and TB-LAMP offer rapid results and can detect drug resistance, which this system cannot. However, the cost and infrastructure requirements of these molecular methods may limit their widespread adoption in high-burden, low-resource settings. Thus, this automated system could potentially bridge the gap between conventional microscopy and molecular methods. It offers improved sensitivity over manual microscopy while being more cost-effective and easier to implement than molecular tests. In a tiered diagnostic approach, this system could serve as an enhanced initial screening tool, with positive or uncertain results then confirmed by molecular methods.

Looking forward, further research is needed to validate these results in diverse settings and populations. Multi-center studies comparing this system directly with both manual microscopy and molecular methods in terms of diagnostic accuracy, cost-effectiveness, and operational feasibility would be valuable. Additionally, exploring the potential of this technology for other diseases requiring microscopic examination could broaden its impact on global health.

Lastly, this automated intelligent microscopy system represents a significant advancement in TB diagnostics. By combining the traditional method of sputum smear microscopy with cutting-edge AI and deep learning technologies, it offers a promising solution to enhance TB detection, particularly in high-burden, resource-limited settings. As we strive to meet the ambitious goals set by the UN High-Level Meeting on TB, innovations like this may play a crucial role in improving TB case detection, reducing diagnostic delays and ultimately assisting in our global efforts to end the TB epidemic.

## Figures and Tables

**Table 1:** Performance of automation system and manual smear microscopy to detect AFBs (before discrepancy resolution by gold standard).

Test Performance		AFB Record by Technicians	
		Positive	Negative
TB-Scan	Positive	177	33
	Negative	17	773

**Table 2:** Performance of the automation system and manual smear microscopy to detect AFBs (after discrepancy resolution by the gold standard).

Test Performance		Gold Standard	
		Positive	Negative
TB-Scan	Positive	194	16
	Negative	17	773

## Data Availability

Validation image dataset can be found at “Lin, Y.E.; Wellgen Medical, (2024), “Detecting Mycobacterium tuberculosis Bacilli from TB Smear Images”, Mendeley Data, V1, doi:10.17632/yhybmfrmgs.1”.
